# Supplementary material for: Reconstructing the ecology of a Jurassic pseudoplanktonic raft colony
Source: R Soc Open Sci. 2020 Jul 22;7(7):200142. doi: 10.1098/rsos.200142 (PMC7428219; doi:10.1098/rsos.200142)
Supplement: Supplementary Information [file rsos200142supp1.docx]

**Supplementary Information: Reconstructing the ecology of a Jurassic psedoplanktonic megaraft colony**

**Introduction to Crinoid Ecology**

The crinoids that are the focus of this study are a wonder of the Mesozoic with as many as 100 individuals covering oyster-encrusted logs up to 14m long (electronic supplementary material, figure S1). The crinoids that inhabited these communities belong to distinct genera with a characteristic morphology. Although a number of unique adaptations have been suggested for these animals, uncertainty exists whether or not this mode of life was possible. Our study is consistent with the special adaptations that these crinoids have, such as distally tapering column, a strengthened attachment structure and the development of an enlarged crown, which does not need to close hermetically in *Seirocrinus*, and a very large crown and high densities of cirri allowing for the enlargement of the cup and the food groove in *Pentacrinites*. Never again do crinoids develop such adaptations to an unusual ecosystem. Although risky, this adaptation allowed these crinoids to spread across Eurasia inhabiting regions as widespread as Alaska and Japan (Hunter and Zonneveld 2008, Hunter et al. 2011).

# 1. Analysis of the spatial positions of attachment discs along the Holzmaden (G1) crinoid log


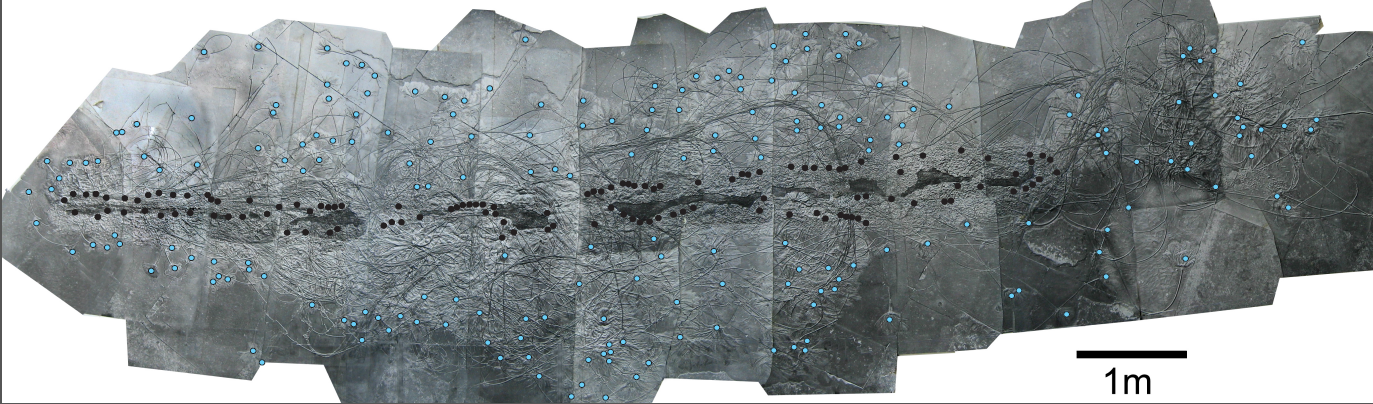


**Figure S1.** The specimen with attachment discs marked in black and heads marked in blue.

## 1. 1 Introduction to SPPA

This species of crinoid are immobile once attached to logs or other substrate (Hagdorn 2016). Therefore, their spatial distributions of their attachment discs on their settled substrate reflect the dispersal processes that brought their larvae to the substrate and the consequent interactions between organisms. As such, analysis of the spatial distributions using spatial point process analyses (SPPA) can shed light on these dispersal process and interactions (e.g. Seidler and Plotkin 2006). SPPA has been effectively applied to the study of modern sessile ecosystems, most notably in terrestrial forests, but also to a limited extent with marine benthic organisms such as barnacles (Lancaster and Downes 2004, Illian et al. 2008).

Comparisons with extant organisms such as barnacles show that when larvae attach to a moving object, such as a boat hull, the spatial distributions are highly anisotrophic - that is they are not uniform across the entire object. Areas which face into the current (such as the bow of a boat) are subject to high turbulence and thus have limited colonization, while the areas sheltered from the current, such as boat sterns, have the highest densities of invertebrate colonizers (Ashton et al. 2014). In contrast when larvae attach to an immobile substrate, their spatial distributions exhibit complete spatial randomness (CSR) when not impacted by environmental variables such as habitat heterogeneities such as patchy rocks (e.g. Edwards and Stachowicz 2011). Comparisons of observed spatial distributions with spatial models can be used to infer the most likely process underlying a spatial distribution. Randomly distributed points, (CSR) can be modelled using homogeneous Poisson processes while anisotrophy can be modelled using heterogeneous Poisson processes. For these heterogeneous Poisson processes, the density of points changes according to a formula, such as with distance along the substrate.

Pair correlation functions (PCFs) are commonly used to describe complex spatial distributions over large distances, where they document how the density of specimens changes with distance (e.g. Wiegand et al. 2007). A CSR (random) population will have a PCF of 1, whereas aggregation is indicated by PCF >1, and segregation by PCF <1. The magnitude of the PCF reflects the intensity of biological and physical processes; a population with PCF=4, for example, is four times more aggregated than one with CSR. If a PCF is significantly non-random, then the specimens have been subject to a biological or ecological process, such as interactions with each other, or their environment. Segregations between specimens occur when organisms cannot overlap with each other, or being within the vicinity of another specimen has a negative effect, while aggregations indicate likely positive effects, such as beneficial substrate, or dispersal induced clustering.

## 1.2 Methods

Data exploration, inhomogeneous Poisson modelling and residual analysis was performed in R using the package spatstat (R core team, 2013, Baddeley et al. 2000, Baddeley et al. 2015). Pair correlation functions (PCFs) were calculated to describe the spatial distributions of discs on the log (electronic supplementary material, figure S1) (Illian et al. 2008). Monte Carlo and Diggle’s goodness-of-fit test5 (the p-value *p_d_*, in which *p_d_* =1 indicates a perfect model fit, and *p_d_* =0 indicates no fit), simulations were used to assess whether the spatial distribution was completely spatially random (Diggle 2003). The PCF value reflects how many times more likely the distribution seen is aggregated (or segregated) compared with CSR. The PCF was plotted (electronic supplementary material, figure S2), and nine hundred and ninety-nine simulations were run to generate simulation envelopes around the CSR. To assess whether the density of the spatial distributions of discs was stronger in any particular direction (that is, it exhibited isotropy); density plots were fit to of the point positions of the discs. To assess how disc density changed along the log, disc density was modelled as a heterogeneous Poisson process dependent on the *x* co-ordinate and then the *y*-co-ordinate. Model fit was assessed using the model residuals (Illian et al. 2008, Wiegand et al. 2007). Model residuals assessed the fit of the model to the data by plotting Q–Q and smoothed residual plots. If the observed line in the Q–Q plot fell outside two standard deviations of the model, the model was rejected (Illian et al. 2008, Wiegand et al. 2007). Akaike information criterion values (Baddeley et al. 2011) were used to compare the relative quality of the statistical models that fitted the data.

## 1.3 Results

The spatial distribution of the discs was found to be significantly segregated (*p_d_* <0.001) below 4.5cm (electronic supplementary material, figure S2). This segregation is hard-core under 2.5cm, which means that no specimens are found within 2.5cm of each other. Between 2.5 and 4.5cm there is a soft-core segregation, which means that while segregation occurs, the likelihood of specimens occurring within this distance is reduced. This result suggests that the attachment discs require a non-overlapping area of 2.5cm, and it is sub-optimal to attach within 2cm of another disc. The density map shows a clear anisotrophy: that is a difference in density dependent on direction, with the highest density on the left hand side, with decreasing density along the log (electronic supplementary material, figure S3). This anisotrophy can be modelled by heterogeneous Poisson model depending on the *x* co-ordinate (electronic supplementary material, Table S1).


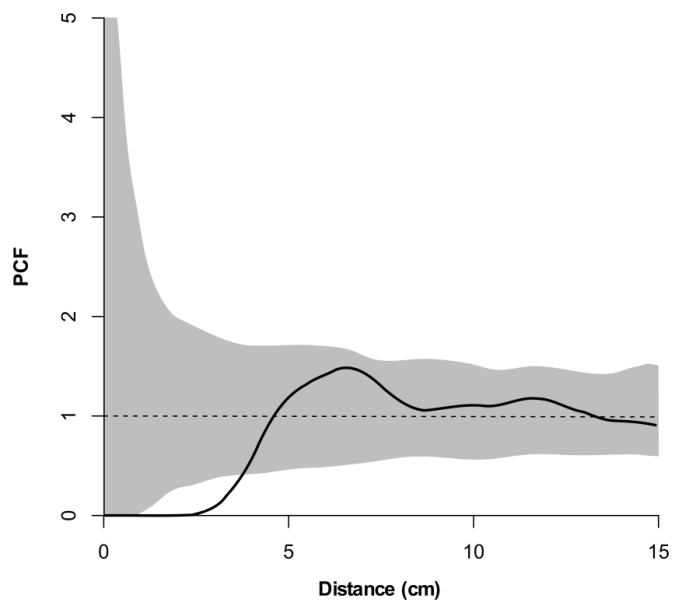


Figure S2. Pair correlation function for attachment discs. The x axis is the inter-point distance between organisms in centimetres. On the y axis, PCF=1 indicates CSR, <1 indicates segregation and >1 indicates aggregation. Grey shaded area depicts the bounds of 99 Monte Carlo simulations of CSR. Since the PCF curve is not completely within these areas, the CSR hypothesis is rejected and one can assume that discs are significantly segregated.


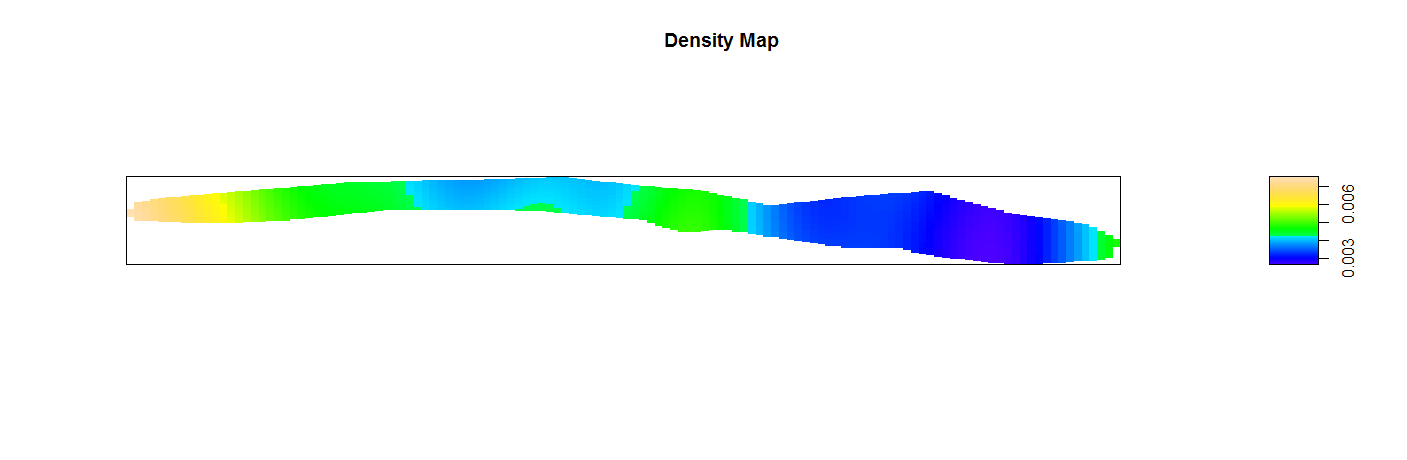


Figure S3. Density map of attachment discs. Note the higher densities on the left hand side.

Table S1. AIC values for different heterogeneous Poisson models dependent on either the x or y co-ordinate. The lower the AIC value, the better fit the model. The lowest AIC value was for the heterogeneous Poisson model dependent on the x coordinate. This best-fit model was within the two standard deviations for the Q-Q plot.

| Model | AIC |
| --- | --- |
| CSR | 1592.544 |
| X | 1591.102 |
| Y | 1594.313 |

# 2. Moisture diffusion model for the fossil wood log (electronic supplementary material*,* figure S1)

Reconstructing water diffusion processes from fossil data is complex due to 1) the difficulty to assign samples reliably to a given taxon (poor preservation of wood structures) and, even if that were possible, 2) the major impact of environmental factors such as humidity or temperature on the diffusion of water in a submerged log.

In Matzke and Maisch (2019, p.98), the authors looked at the same wood sample we selected and suggested three categories for the classification of fossil driftwood depending on levels of encrustation by bivalves and/or crinoids. Thin sections have been possible for only the non-encrusted wood logs (type A), allowing their classification as conifer “*Araucarioxylon*”. However, no classification has been possible for the other log types due to wood structures not being observable.

A more general examination of Early Jurassic vegetation in the Danish Basin suggests a flora composed of tree ferns (*Deltoidospora*) and pinacean conifers (*Pinuspollenites minimus*), along with common taxodiacean/cupressacean conifers, ginkgos/cycads and corystospermous seed ferns ((Lindström, Erlström, Piasecki, Nielsen, & Mathiesen, 2017).

## 2.1 Physical properties of wood

### 2.1.1 Wood types and variety

Trees are divided into two classes: conifers (or softwoods) and broadleaf trees (or hardwoods). The main distinction between the two classes is that hardwoods possess vessel elements in addition to the common wood fibers (or tracheids). Both classes display a broad range of densities with, for example, balsa (hardwood) showing lower densities (0.2*10^3^ kg/m^3^) than softwoods and yew (softwood) being denser (0.7*10^3^ kg/m^3^) than many hardwoods.

### 2.1.2 Structures

Cross sections for hardwoods and softwoods reveal dark and light areas that correspond to latewood and earlywood respectively. Each area is composed of tubular structures called wood fibers or tracheids that are around 30 μm in diameter and 3 mm in length ([Jacobson 2006](#_ENREF_2)). The main structural distinction between softwood and hardwood is the existence of additional specialized vessels in the latter through which moisture is conducted. These vessels present a variety of features (presence or absence of perforations, size of the perforations, lignin reinforcements, etc.) and diameters. There is currently no definitive model that would consider the chemical and physical processes involved in moisture transport in wood and the phenomenon is likely to be affected by many variables simultaneously ([Skaar 1984](#_ENREF_7); [Wood et al. 2002](#_ENREF_10)).

### 2.1.3 Composition

Wood consists of three polymers: cellulose, hemicellulose and lignin. These polymers are not evenly distributed as cellulose amounts to between 40 and 50% of the material in mass, depending on the species ([Sjostrom 1993](#_ENREF_6)). The remaining mass is roughly evenly divided between hemicelluloses and lignin. The three polymers assemble to form microfibrils that are typically 5000 nm long and 10-20 nm wide. In turn, these microfibrils are arranged in sheets that constitute the cell wall of wood fibers and the relative thickness of these sheets is also species-specific. The varying abundance of the polymers coupled with their distinct chemical properties explain the complex sorption behaviour of wood materials.

### 2.1.4 Wood Decay

It is unknown whether the log structures in this study were gymnosperms or angiosperms as the wood is not preserved; additionally, the variety of gymnosperm species themselves are critical to the long term survival of the raft. Generally recent gymnosperm conifer forests produce much more wood debris than angiosperm forests, therefore we can assume there was plenty of available material. The log structures clearly survived high-energy fluviatile environment or entered the system from the margins of the epicontinental sea. From this, we can infer that a log from the Jurassic gymnosperm forest in a swamp, estuary or delta was dislodged and floated out to sea where bivalves and crinoid larval disks attached and formed a complex community structure. Fresh water fluviatile environments have lower rates of microbial decay in streams compared to the open ocean; conversely these environments are home to elevated amounts of fungus and terrestrial invertebrates such as insects. The latter could not survive in the open ocean environment finding it intolerable. It is often cited that log structures are less common in the open ocean due the presence of marine invertebrates that would break down the wood structures; however, our examples were likely protected by the anoxia prevalent in both the Holzmaden and in the Lias may have meant few invertebrates could have infiltrated the log. Additionally, many of the agents found in the modern oceans that break down floating wood (e.g. driftwood talitrids) evolved post mid-Jurassic. In terms of properties of the wood itself, the initial density can influence decay rates. Our idealized models assume that the density of wood was the same across the log; close to the composition of sapwood that contains functioning vascular tissues. Therefore spaces are likely to absorb water and decay at a higher rate. Gymnosperm tracheids (pore spaces) are much smaller than angiosperms vessels. The density of these spaces in gymnosperm wood declines higher up the tree structure which means the low density structures further up the log would have been better candidates for longer lasting wood megarafts. This sapwood is surrounded by outer bark, inner bark which includes the phloem and core consisting of the heartwood. With a much lower rate of absorption the presence of plentiful heartwood would have strengthened the viability of the system considerably. Gymnosperm sapwood contains much less living tissue 5-11 % as opposed to 11-48 % found in angiosperms. The lack of living tissue would have meant a much lower decay rate in this type of wood.

## 2.2 Moisture sorption model

### 2.2.1 Diffusion coefficients

The diffusion of gas occurs within the lumen of cells and is dependent on the diffusivity of water vapor in air, temperature and the saturated vapor pressure. The diffusion of bound water is dependent on the temperature and the moisture content. These two values can be combined to reflect the general diffusion coefficient for both radial (*Dr*) and longitudinal (*Dl*) moisture transfers ([Baronas et al*.* 2001](#_ENREF_1)).

### 2.2.2 Moisture movement in wood

The model considers the diffusion of moisture within a cylinder of revolution of radius *r* and length *L*. Wood vessels are running along the length of the cylinder and are orthogonal to the circular cross section.

The diffusion of moisture can be modeled as a Fickian phenomenon by the equation (1) that is set in a one-dimension (longitudinal) system without convection.

|  | $\frac{\partial u}{\partial t}=D\frac{\partial^{2}u}{\partial x^{2}}$ |  |
| --- | --- | --- |

In a cylindrical system, the equation should be adapted to reflect the diffusion that happens both longitudinally and radially:

|  | $\frac{\partial u}{\partial t}=D_{r}\left[ \frac{1}{r}\frac{\partial}{\partial r}\left( r\frac{\partial u}{\partial r} \right) \right]+D_{l}\frac{\partial^{2}u}{\partial x^{2}}$ |  |
| --- | --- | --- |

##### Initial conditions

u(0,r,t)=u(L,r,t)=100; u(x,b,t)=100;

### 2.2.3 Longitudinal component

If we employ a classic separation method, we can express u_l_(r,x) as a product of two functions X and T such as:

|  | $u_{l}\left( r,x \right)=X\left( x \right)T(t)$ |  |
| --- | --- | --- |

The longitudinal component of equation (2) becomes:

|  | $\frac{1}{D_{l}}\frac{T^{'}}{T}=\frac{X^{"}}{X}=-\mu$ |  |
| --- | --- | --- |

If we consider the spatial component of this equivalence first, we can say that μ>0 is the only condition leading to non-trivial solutions:

|  | $X_{n}\left( x \right)=A_{n}\sin\left( \frac{n\pi x}{L} \right)$ |  |
| --- | --- | --- |

with μ=(nπ/L)^2^ . By injecting this value into the temporal component, we obtain:

|  | $T_{n}^{'}+\frac{D_{l}n^{2}\pi^{2}}{L^{2}}T_{n}=0$ |  |
| --- | --- | --- |

which leads to:

|  | $T_{n}\left( t \right)=B_{n}exp\left( \frac{-D_{l}n^{2}\pi^{2}}{L^{2}}t \right)$ |  |
| --- | --- | --- |

By combining (5) and (7) and summing all the components, we obtain a general expression for the moisture profile along the longitudinal axis:

|  | $u_{l}\left( x,t \right)=100-\sum_{n=1}^{\infty} C_{n}\sin\left( \frac{n\pi x}{L} \right)exp\left( \frac{-D_{l}n^{2}\pi^{2}}{L^{2}}t \right)$ |  |
| --- | --- | --- |

the value of C_n_ is fixed by the initial condition at t=0 and the boundary conditions at x= L:

|  | $C_{n}=\frac{2}{L}\int_{0}^{L} \varphi(x)\sin\left( \frac{n\pi x}{L} \right)dx$ |  |
| --- | --- | --- |

For example, if L=π and u_l_(x,0)=-x(π-x),

|  | $C_{n}=\frac{2}{L}\int_{0}^{\pi} x\left( \pi-x \right)\sin\left( nx \right)dx$ |  |
| --- | --- | --- |
| and | $C_{n}=4\frac{1-\left( -1 \right)^{n}}{n^{3}\pi}$ |  |

This initial distribution of the moisture is a good approximation of a moisture profile.

In the end, the longitudinal diffusion of moisture in a cylinder of length π can be expressed as:

|  | $u_{l}\left( x,t \right)=100-\frac{8}{\pi}\sum_{n=1}^{\infty} \frac{\sin\left( \left( 2n-1 \right)x \right)}{\left( 2n-1 \right)^{3}}exp\left( -D_{l}\left( 2n-1 \right)^{2}t \right)$ |  |
| --- | --- | --- |
| with | $D_{l}=\frac{\sqrt{por}D_{b}D_{v}}{\left( 1-por \right)\left( \sqrt{por}D_{b}+\left( 1-\sqrt{por} \right)D_{v} \right)}$ |  |

where *por* is the porosity of the material (which is a function of dry density), *Db* is the diffusion coefficient for bound liquid water and *Dv* is the diffusion coefficient for water vapour. Formulas to calculate Db and Dv are taken from Baronas (2001) and Krabbenhoft (2003) for fully saturated conditions (i.e. green wood).

### 2.2.4 Radial component

A similar approach can be taken with the radial component of the moisture function.

|  | $\frac{\partial u_{r}}{\partial t}=D_{r}\left( \frac{\partial^{2}u}{\partial u^{2}}+\frac{1}{r}\frac{\partial u}{\partial r} \right)$ |  |
| --- | --- | --- |

A separation of the variables leads to:

|  | $u_{r}\left( r,t \right)=R\left( r \right)T(t)$ |  |
| --- | --- | --- |

and thus equation (12) can be re-written as:

|  | $\frac{1}{D_{r}}\frac{1}{T}\frac{\partial T}{\partial t}=\frac{1}{R}\left( \frac{\partial^{2}R}{\partial r^{2}}+\frac{1}{r}\frac{\partial R}{\partial r} \right)=-\left( \frac{m}{b} \right)^{2}$ |  |
| --- | --- | --- |

The only non-trivial solutions for equation (14) are for a separation constant that is negative (-(m/b)^2^) and this leads to:

|  | $R\left( r \right)=J_{0}\left( \frac{m}{b}r \right)$ |  |
| --- | --- | --- |

If we focus now on the temporal element of the moisture function, the boundary condition R(b)=0 suggests to consider all the possible solutions of the equation J_0_(m_n_)=0

The equation (14) becomes then:

|  | $\frac{\partial T_{n}}{\partial t}+\left( \left( \frac{m_{n}}{b} \right)^{2}D_{r} \right)T_{i}=0$ |  |
| --- | --- | --- |
| and thus | $T_{n}(t)=M_{n}exp\left( {{-\left( \frac{m_{n}}{b} \right)}^{2}D}_{r}t \right)$ |  |

By combining (15) and (17) and summing all the components, we obtain a general expression for the moisture profile along the longitudinal axis:

|  | $u_{r}\left( r,t \right)=\sum_{n=1}^{\infty} M_{n}J_{0}\left( \frac{m_{n}}{b}r \right)exp\left( {{-\left( \frac{m_{n}}{b} \right)}^{2}D}_{r}t \right)$ |  |
| --- | --- | --- |

The boundary condition u_r_(r,0)=M_init_ leads to:

|  | $M_{n}=\frac{\left( 2M_{init} \right)}{m_{n}J_{1}(m_{n})}$ |  |
| --- | --- | --- |

and therefore:

|  | $u_{r}\left( r,t \right)=2M_{init}\sum_{n=1}^{\infty} \frac{1}{m_{n}J_{1}(m_{n})}J_{0}\left( \frac{m_{n}}{b}r \right)exp\left( {{-\left( \frac{m_{n}}{b} \right)}^{2}D}_{r}t \right)$ |  |
| --- | --- | --- |

At water saturation, *Dr* can be approximated as a fraction of *Dl* as permeabilities *Kr* and *Kl* have been experimentally correlated to the saturation level of the wood *S*: $\frac{K_{l}}{K_{r}}={10}^{4}S^{8}$ (Krabbenhoft, 2003).

### 2.2.5 Weight of the crinoid colony

Part of our calculation is to seek to understand the total weight of the colony. We take into account the following factors:

1. The population is always preserving optimum intrinsic growth parameters.
2. No environmental factor limits the growth of the community during the duration of the simulation.
3. There is no significant water absorption within the oysters or the crinoids during the growth of the colony.

We conclude that the mass of the community in the most extreme circumstances could reach around 880 kg for a log that is 10 m long and 0.4 m in diameter with a carrying capacity of 1000 individuals per square meter. If the carrying capacity is less than 600 individuals per square meter, only logs with a density higher than 0.5 (pine is around 0.5) would manage to sink within a 20-year window and the community would then weigh around 630 kg. As for the mass of the log a Shortleaf pine (softwood) with a kiln dry specific gravity of 0.47 (average for softwood), the green wood will have a general mass varying between 950 and 1100 kg for a log that is 10 m long and 40 cm in diameter. This could be regarded as a minimum value with modern hardwood logs up to 15,000 kg. That means that the weight of the crinoid community in that optimum growth scenario (super high carrying capacity) matches almost the original mass of a softwood log. However these high carrying capacities >200 hundred crinoids per square meter are rare and it is likely that the crinoid community mass did not reach 600 kg for a span of 20-50 years on our large logs.

## 2.3 Results

The sorption model considers wooden logs to be preserved all throughout the soaking process and it assumes that the material is homogenous. Therefore, the model likely overestimates the longevity of the log structure but it allows to establish a maximum window of time for the crinoid community to develop. When green, wood displays moisture contents that can reach beyond 150% (e.g. Western Redcedar at 249% or old growth Redwood at 210%) but these ranges have not been modelled for this study.


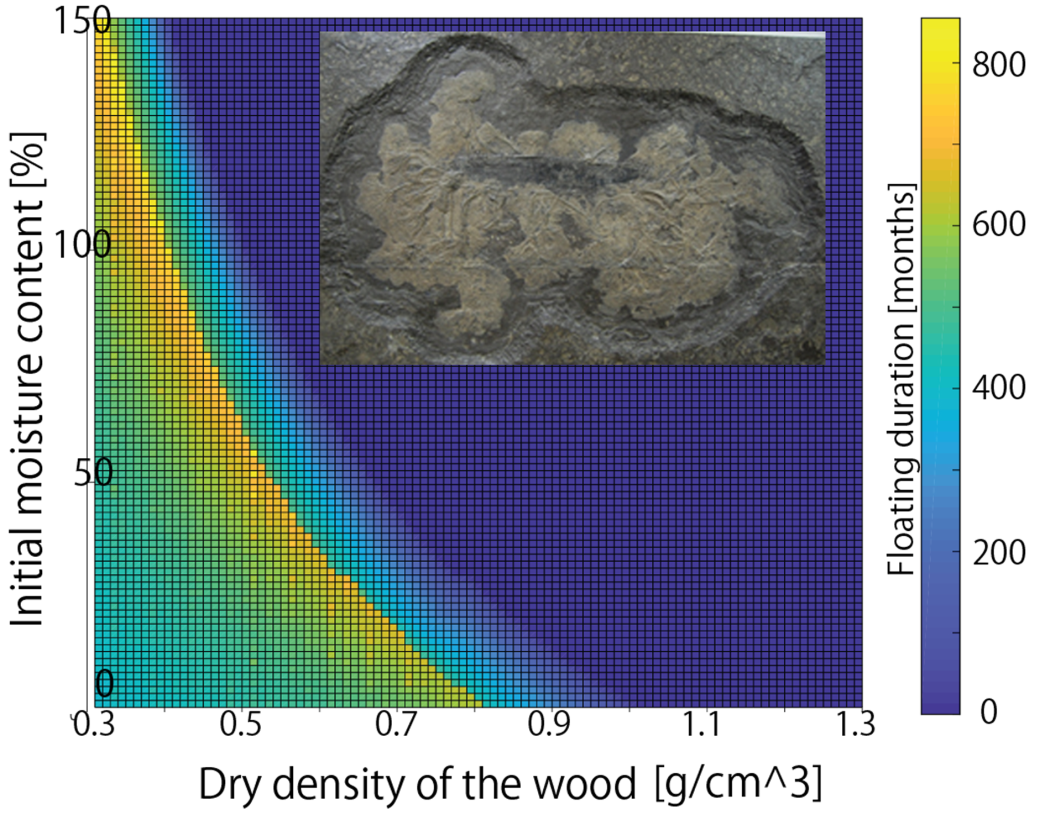


Figure S4: Floating duration (in months) until sinking for small colonies [Göttingen (S1) and Stuttgart 1 (S2) – 40 cm long Diameter 5-6 cm (Community Removed); see Figure 3a] as a function of the dry density (g/cm^3^) of the material relative to water and its initial moisture content (%). In green wood, diffusion of moisture occurs longitudinally and radially (with a tangential component) for water vapour, liquid water bound to cell walls, and liquid water running free through vessels and tracheids . Initial moisture content reflects the humidity of the surrounding atmosphere (relative humidity) as well as its temperature, and moisture content constrains the diffusion coefficients. In green wood, moisture content can vary between sapwood (>100% where sap flows) and heartwood (~40% closer to the pith) but the model assumes an average moisture content throughout the log. The log will sink as soon as the system {wood log + water} is denser than water. For example (red dotted lines), a log with a dry density of 0.4 g/cm^3^ and an initial moisture content of 30% (fiber saturation point) would stay afloat for approximately 400 months or 33 years.


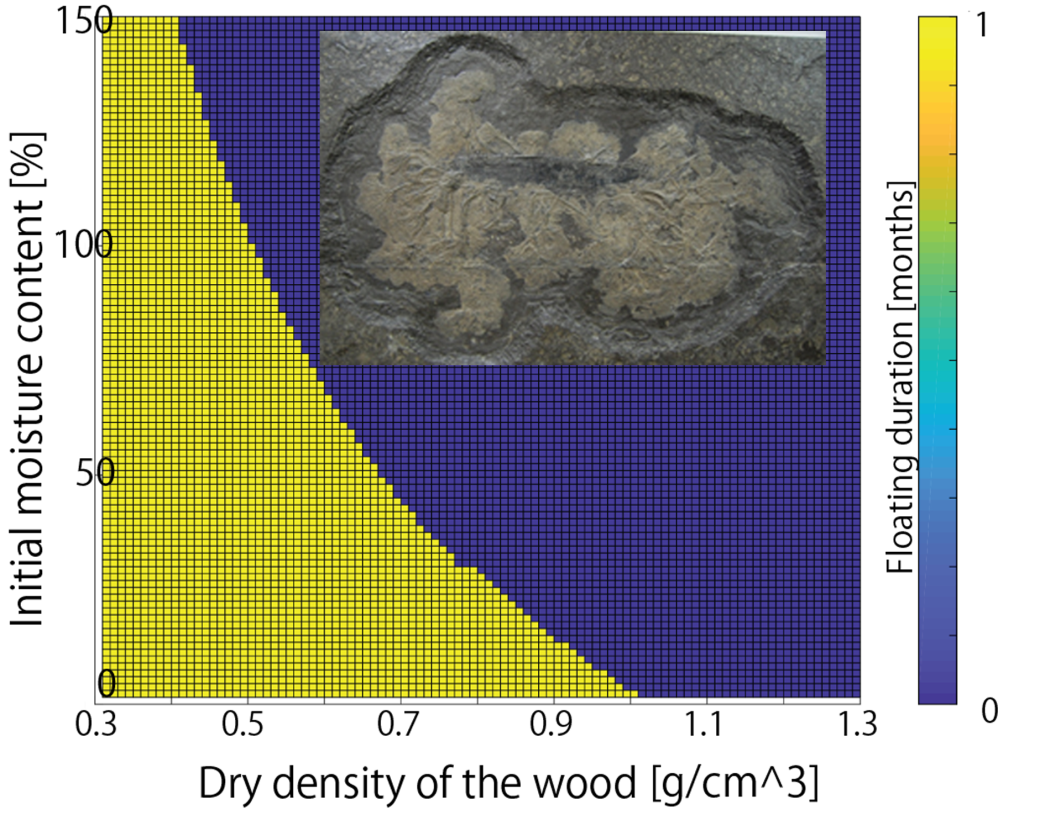


Figure S5: Floating duration (in months) until critical mass for small colonies [Göttingen (S1) and Stuttgart 1 (S2) – 40 cm long Diameter 5-6 cm (Community Included); see Figure 3b] as a function of the dry density (g/cm^3^) of the material relative to water and its initial moisture content (%). In addition to the soaking process, we have loaded the log with a community of crinoids with an arbitrary individual growth rate extrapolated from extant species of *Neocrinus decorus*, *Endoxocrinus parae* and *Cenocrinus asterius* within [0.6-17.0 cm/yr] (Messing *et al*. 2007). Monthly growth rates were converted into mass increments using the specific gravity of calcite at 2.7g/cm^3^. The log will sink as soon as the system {wood log+water+crinoids} is denser than water. In this case, the log sinks at the end of the first monthly increment of growth.


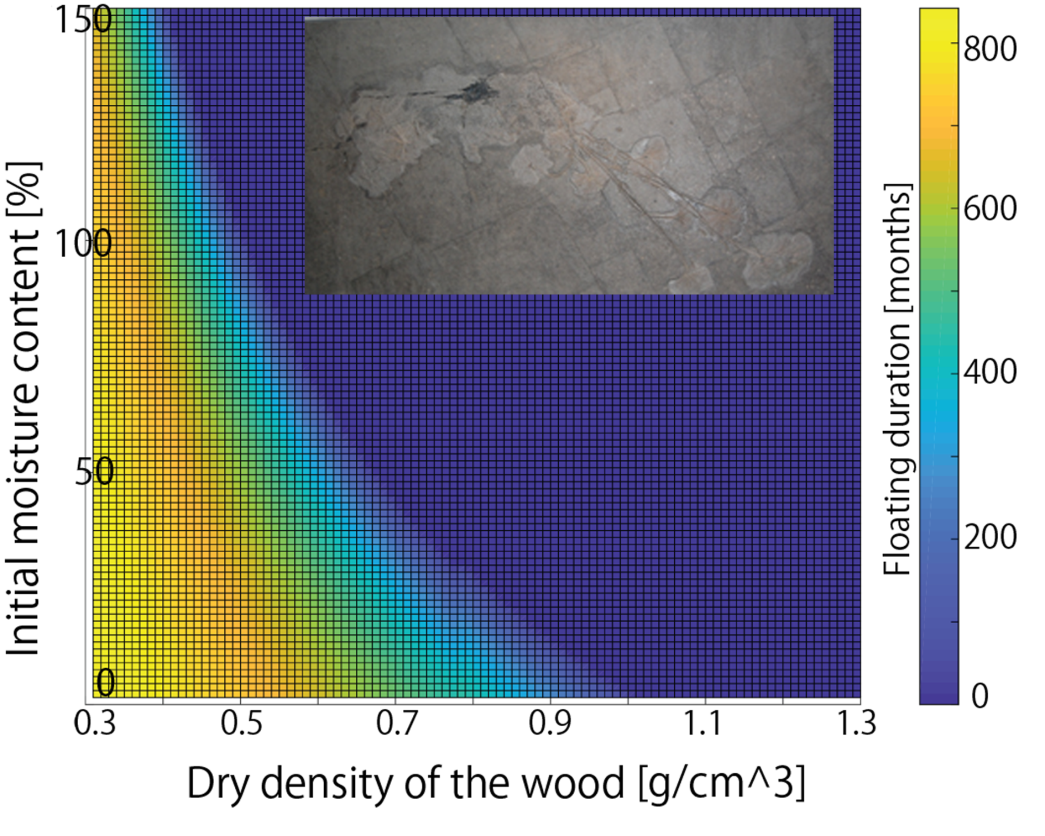


Figure S6: Floating duration (in months) until critical mass for medium colonies [Frankfurt (M1) and Dotternhausen (M2) – 190 cm long, Diameter 8-12 cm (Community Removed); see Figure 3c] as a function of the dry density (g/cm^3^) of the material relative to water and its initial moisture content (%). For example, a wood of dry density 0.4 g/cm^3^ and an initial moisture content of 30% (fiber saturation point) would take approximately 600 months or 50 years (red dotted lines).


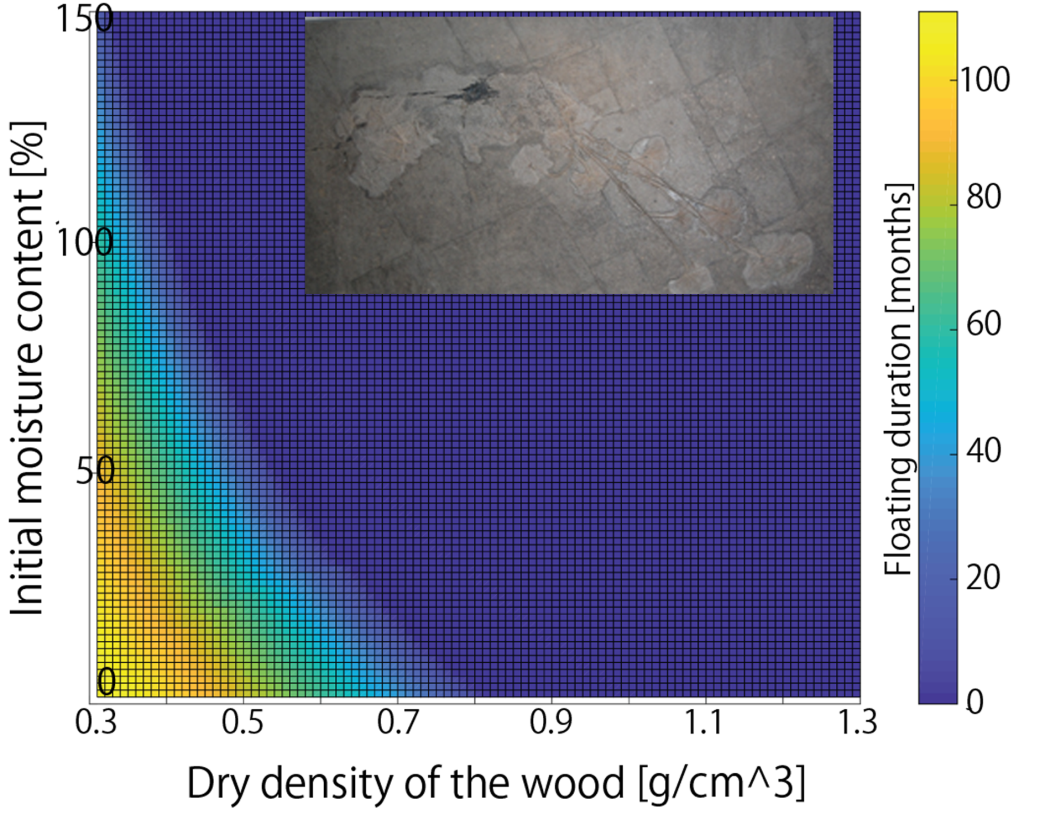


Figure S7: Floating duration (in months) until critical mass for small colonies [Frankfurt (M1) and Dotternhausen (M2) – 190 cm long, Diameter 8-12 cm (Community Included); see Figure 3d] as a function of the dry density (g/cm^3^) of the material relative to water and its initial moisture content (%).


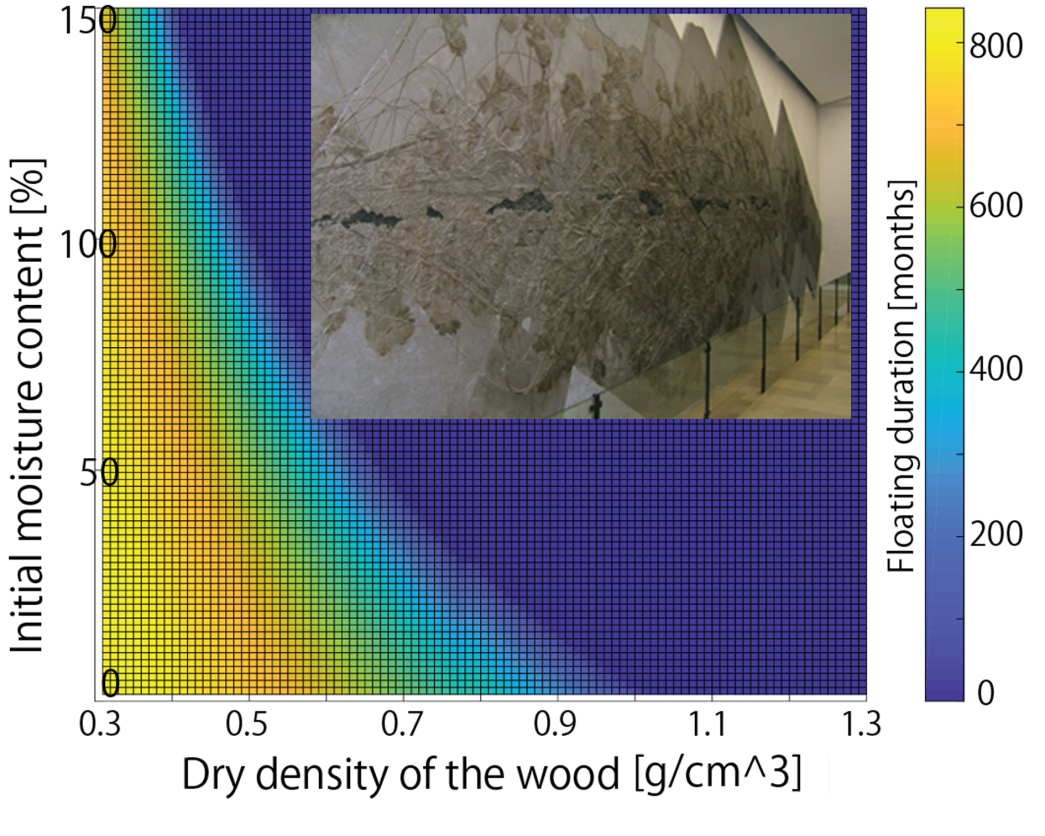


Figure S8: Floating duration (in months) until critical mass for medium colonies [Holzmaden (G1) and Stuttgart 2 (G2) – 12 m long Diameter 25-28 cm (Community Removed); see Figure 3e] as a function of the dry density (g/cm^3^) of the material relative to water and its initial moisture content (%). For example, at fiber saturation (30% moisture content), a material of dry density of 0.4 g/cm^3^ would take approximately 800 months or 66 years (red dotted lines).


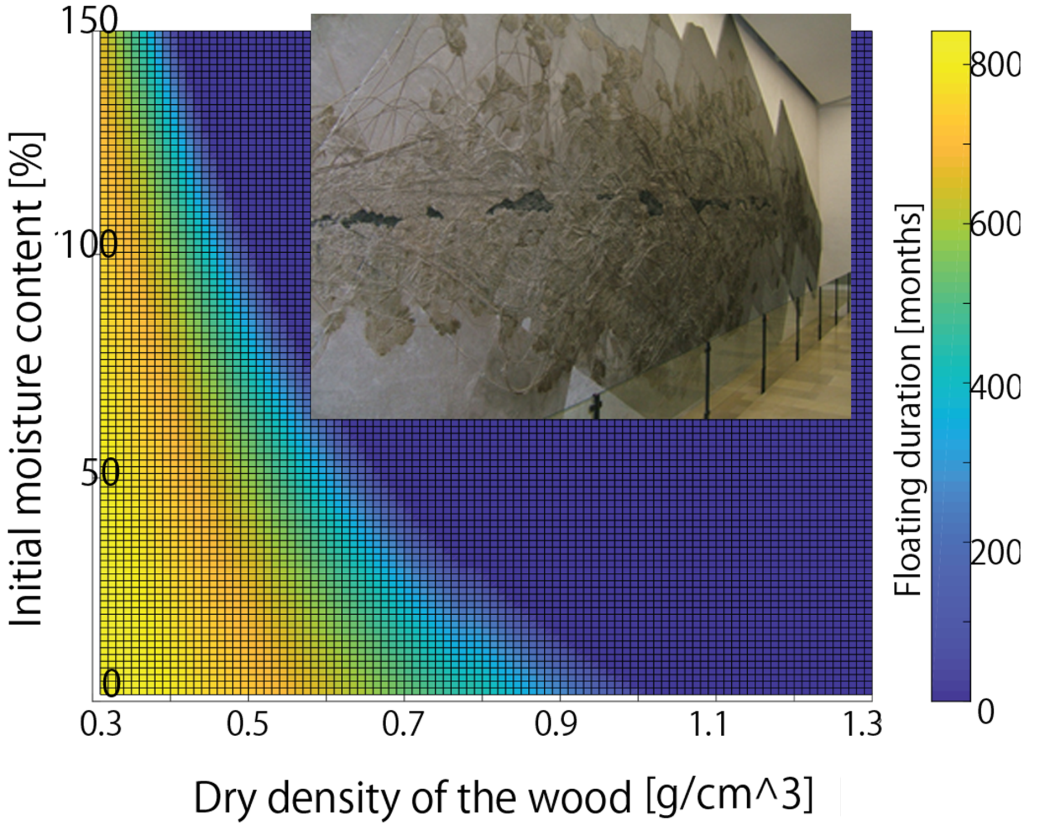


Figure S9: Floating duration (in months) until critical mass for massive colonies [Holzmaden (G1) and Stuttgart 2 (G2) – 12 m long Diameter 25-28 cm (Community Included); see Figure 3f] as a function of the dry density (g/cm^3^) of the material relative to water and its initial moisture content (%).

.

# 3 *Pseudomytilus dubius* community growth model

## 3.1 Introduction

The population model incorporated both the spatial distribution along the log as well as life-history estimates (fecundity, mortality, settling rates and maturation time) for a complete life cycle of *Pseudomytiloides dubius* (Sowerby, 1829). A life cycle was defined as the duration from the release of eggs, through the planktonic larval stage, settlement and growth to reach a mature stage allowing the release of the next generation of eggs. Simulations for the growth of *P. dubius* communities were conducted using the mathematical package MATLAB R2017a.

The log surface was sectioned into 10 compartments (or boxes) and the model quantitatively estimated the success (settlement and growth) of a population within a given box (Kanary et al. 2011). The model was initialized by assuming one mature adult in box 1 and it estimated the number of mature oysters at each following time increment (one year) across the log (10 boxes).

## 3.2 Growth model

Concerning *Pseudomytiloides dubius*, Caswell and Coe (2013) suggest a similar life cycle as *Mulinia lateralis* (opportunistic species, 2 years max, sexual maturity at 2 months) rather than *Mya arenaria* (equilibrium species, 20 years max, sexual maturity at 2 years). For the model, the growth pattern of *P. dubius* was extrapolated from large scale regression analyses conducted on bivalve morphologies (Rigdway Richardson and Austad 2011).

The model assumed that once settled, an individual does not change box (Raillard & Ménesguen 1994) and the time to reach maturity is within 1 year (Okaniwa, Miyagi, Sasaki & Tanabe 2010). In a population, only a certain percentage is actually spawning (rSpawn) which leads these mature adults to release eggs (per capita reproductive rate rRep). Of these released eggs, only a fraction of the larvae can settle (sRate). The further the box is from the spawning location, the less likely the larva will settle and a simple transition matrix tP(i,n) was designed to illustrate this differential implantation distribution. Each entry in the transition matrix tP(i,n) represents the probability of transitioning from one compartment to another through simple dispersal by water currents because the average swimming speed of a veliger larva only ranges between 10^-4^ and 4*10^-3^ m/s (Sprung 1984). These assumptions allowed providing a simplified frame for the growth model.

The population growth process can thus be expressed as:

|  | $X_{(i,t)}=\left( 1-r_{Mort} \right)X_{(i, t-1)}+\sum_{n=1}^{10} r_{Spawn}r_{Rep}s_{Rate}\left( {tP}_{(i,n)}*X_{(n,t-1)} \right)$ | (22) |
| --- | --- | --- |

With

|  | $X_{i,t}=min\left\{ X_{i,t}, K \right\}$ | (23) |
| --- | --- | --- |

Where K is the maximum carrying capacity of the log compartment (individuals/ m2). Carrying capacity is understood as a simple saturation function: at each time step, if the value of X computed by equation 1 is larger than K, it is truncated to K because additional larvae cannot settle on a log compartment once the carrying capacity has been reached. In addition, n is the index associated to the destination compartment.

Spawning rate, per capita reproductive rate and mortality rate correspond to average values taken from the literature about *Mytilus* or Mytiloidea (Petrovic & Guichard 2008; Stoeckel et al. 2004) or *Crassostrea virginica* (Powell et al. 1996; Wang et al. 2008). The values for the parameters appear in Table S2.

Table S2: Values of the parameters retained for the model of population growth. Values are extracted from existing literature about Mytiloidea communities (Petrovic & Guichard 2008; Stoeckel et al. 2004) and oyster populations in the USA (Powell et al. 1996; Wang et al. 2008).

| **Parameter** | **Value** | **Reference** |
| --- | --- | --- |
| Mortality rate (*rMort*) | 0.3 | ([Petrovic](file:///C:\Users\ookab\Downloads\Pseudomytilus%20dubius%20population%20growth%20model.docx#_ENREF_1) & Guichard 2008) |
| Spawning rate (*rSpawn*) | 0.15 | ([Wang *et al.* 2008](file:///C:\Users\ookab\Downloads\Pseudomytilus%20dubius%20population%20growth%20model.docx#_ENREF_7)) |
| Per capita reproductive rate (*rRep*) | 500 | ([Stoeckel](file:///C:\Users\ookab\Downloads\Pseudomytilus%20dubius%20population%20growth%20model.docx#_ENREF_1) et al. 2004) |
| Settlement rate (*sRate*) | 0.10 | ([Powell *et al.* 1996](file:///C:\Users\ookab\Downloads\Pseudomytilus%20dubius%20population%20growth%20model.docx#_ENREF_5)) |
| Maximum carrying capacity (*K*) | 50~15000 ind./m^2^ | (Caswell and Coe 2013) |

The model allowed to reconstruct the number of years required to reach sinking density for the whole log for various values of K (Table S3). In some cases, the dimensions of the log combined with the density of the wood material would allow the loaded log never to reach sinking threshold within the 20-year window (x). The range for K is extracted from Caswell and Coe (2013).

Table S3: Number of years required for a growing population of oysters to sink a sealed wood log (no soaking). In this simulation, the wood log had a constant geometry with a length of 10 m and a radius of 0.2 m, corresponding roughly to the dimensions of the Holtzmaden log. “x” marks indicate that no sinking occurred within the computation window of 20 years.

|  | | Maximum carrying capacity (population density in 1000 ind/m2) | | | | | | | | | | | | | | | | | | | |
| --- | --- | --- | --- | --- | --- | --- | --- | --- | --- | --- | --- | --- | --- | --- | --- | --- | --- | --- | --- | --- | --- |
|  |  | 0.05 | 0.2 | 0.3 | 0.4 | 0.5 | 1 | 2 | 3 | 4 | 5 | 6 | 7 | 8 | 9 | 10 | 11 | 12 | 13 | 14 | 15 |
| Wood density | 0.3 | x | x | x | x | x | x | 19 | 17 | 16 | 16 | 15 | 15 | 15 | 15 | 15 | 14 | 14 | 14 | 14 | 14 |
|  | 0.4 | x | x | x | x | x | x | 18 | 17 | 16 | 15 | 15 | 15 | 15 | 14 | 14 | 14 | 14 | 14 | 14 | 14 |
|  | 0.5 | x | x | x | x | x | x | 17 | 16 | 15 | 15 | 15 | 14 | 14 | 14 | 14 | 14 | 14 | 14 | 13 | 13 |
|  | 0.6 | x | x | x | x | x | 19 | 17 | 15 | 15 | 14 | 14 | 14 | 14 | 14 | 13 | 13 | 13 | 13 | 13 | 13 |
|  | 0.7 | x | x | x | x | x | 18 | 16 | 15 | 14 | 14 | 14 | 13 | 13 | 13 | 13 | 13 | 13 | 13 | 13 | 13 |
|  | 0.8 | x | x | x | x | 19 | 16 | 14 | 14 | 13 | 13 | 13 | 13 | 12 | 12 | 12 | 12 | 12 | 12 | 12 | 12 |
|  | 0.9 | x | x | 18 | 17 | 16 | 14 | 13 | 12 | 12 | 12 | 12 | 12 | 11 | 11 | 11 | 11 | 11 | 11 | 11 | 11 |
|  | 1.0 | 3 | 3 | 3 | 3 | 3 | 3 | 3 | 3 | 3 | 3 | 3 | 3 | 3 | 3 | 3 | 3 | 3 | 3 | 3 | 3 |

## References

Ashton G, Davidson I, & Ruiz G (2014) Transient small boats as a long-distance coastal

vector for dispersal of biofouling organisms. *Estuaries and Coasts* 37(6):1572–1581.

Baddeley A, Rubak E, & Turner R (2015) Spatial Point Patterns: *Methodology and*

*Applications with R* (CRC Press).

Baddeley A & Turner R (2000) Practical Maximum Pseudolikelihood for Spatial Point

Patterns: (with Discussion). *Australian & New Zealand Journal of Statistics* 42(3):283–322.

Baddeley A, Rubak E, & Møller J (2011) Score, pseudo-score and residual diagnostics for

spatial point process models. *Statistical Science* 26(4):613–646.

Baronas R, Ivanauskas F, Juodeikiene I, & Kajalavicius A (2001) Modelling of moisture

movement in wood during outdoor storage. *Nonlinear Analysis: Modelling and Control* 6(2):3–14.

Brown SN (2011) *Ecology and enhancement of the flat oyster Ostrea chilensis (Philippi,*

*1845) in central New Zealand.* Doctor of Philosophy (University of Canterbury, New Zealand).

Caswell, B. A., & Coe, A. L. (2013). Primary productivity controls on opportunistic bivalves

during Early Jurassic oceanic deoxygenation. *Geology*, *41*(11), 1163-1166.

Diggle PJ (2003) *Statistical Analysis of Spatial Point Patterns* (Academic Press, San Diego)

2nd Ed.

Edwards KF & Stachowicz JJ (2011) Spatially stochastic settlement and the coexistence of

benthic marine animals. *Ecology* 92(5):1094–1103.

Hagdorn H (2016) From benthic to pseudoplanktonic life: morphological remodeling of the

Triassic crinoid Traumatocrinus and the Jurassic Seirocrinus during habitat change. *PalZ* 90(2):225–241.

Hunter AW, Oji T, & Okazaki Y (2011) The occurrence of the pseudoplanktonic crinoids

Pentacrinites and Seirocrinus from the Early Jurassic Toyora Group, western Japan. *Paleontological research* 15(1):12–22.

Hunter AW & Zonneveld J-P (2008) Palaeoecology of Jurassic encrinites: reconstructing

crinoid communities from the Western Interior Seaway of North America. *Palaeogeography, Palaeoclimatology, Palaeoecology* 263(1):58–70.

Illian J, Penttinen A, Stoyan H, & Stoyan D (2008) *Statistical Analysis and Modelling of*

*Spatial Point Patterns* (John Wiley & Sons, Chichester) p 560.

Jacobson A (2006) *Diffusion of chemicals into green wood*. (Georgia Inst. of Technology).

Jacobson AJ & Banerjee S (2006) Diffusion of tritiated water into water-saturated wood

particles. *Holzforschung* 60(1):59–63.

Kanary L, Locke A, Watmough J, Chassé J, Bourque D & Nadeau A (2011). Predicting larval

dispersal of the vase tunicate *Ciona intestinalis* in a Prince Edward Island estuary using a matrix population model. *Aquatic invasions*, *6*, 491-506.

Krabbenhøft K, Damkilde L, & Hoffmeyer P (2004) *Moisture transport in wood: A study of*

*physical-mathematical models and their numerical implementation.* Doctor of Philosophy (University of Denmark).

Lancaster J & Downes B (2004) Spatial point pattern analysis of available and exploited

resources. *Ecography* 27(1):94–102.

Lindström S, Erlström M, Piasecki S, Nielsen LH, & Mathiesen A (2017). Palynology and terrestrial ecosystem change of the Middle Triassic to lowermost Jurassic succession of the eastern Danish Basin. *Review of Palaeobotany and Palynology*, 244, 65–95.

MATLAB R2017a. MATLAB and Statistics Toolbox Release 2017a, The MathWorks, Inc.,

Natick, Massachusetts, United States.

Matzke AT & Maisch MW (2019). Palaeoecology and taphonomy of a seirocrinus (Echinodermata: Crinoidea) colony from the early jurassic posidonienschiefer formation (early toarcian) of dotternhausen (SW Germany). *Neues Jahrbuch Fur Geologie Und Palaontologie - Abhandlungen*, 291(1), 89–107.

Messing CG, David J, Roux M, Améziane N, Baumiller TK. 2007 In situ stalk growth rates in tropical western Atlantic sea lilies (Echinodermata: Crinoidea). *Journal of Experimental Marine Biology Ecology*, 353, 211-220.

Okaniwa N, Miyaji T, Sasaki T & Tanabe K (2010). Shell growth and reproductive cycle of

the Mediterranean mussel Mytilus galloprovincialis in Tokyo Bay, Japan: relationship with environmental conditions. *Plankton and Benthos Research*. *5*. 214-220. 10.3800/pbr.5.214.

Petrovic F & Guichard F (2008) Scales of Mytilus spp. population dynamics: importance of

adult displacement and aggregation. *Marine Ecology Progress Series 356*: 203-214.

https://doi.org/10.3354/meps07250

Philippi RA (1845) Abbildungen und Beschreibungen Neuer oder Wenig Bekannter

Conchylien (Theodor Fischer, Cassel).

Powell EN, Klinck JM, & Hofmann EE (1996) Modeling diseased oyster populations. II.

Triggering mechanisms for *Perkinsus marinus* epizootics. *Journal of Shellfish Research* 15:141–165.

R Core Team (2013) R: A language and environment for statistical computing. *R Foundation*

*for Statistical Computing*.

Raillard O & Ménesguen A (1994) An ecosystem box model for estimating the carrying

capacity of a macrotidal shellfish system. *Marine Ecology Progress Series* 115:117–130.

Seidler TG & Plotkin JB (2006) Seed dispersal and spatial pattern in tropical trees. *PLoS*

*Biology* 4(11):e344.

Shi SQ (2007) Diffusion model based on Fick’s second law for the moisture absorption

process in wood fiber-based composites: is it suitable or not? *Wood Science and Technology* 41(8):645–658.

Sjostrom E (1993) *Wood Chemistry: Fundamentals and Applications* (Academic Press, San

Diego) 2nd Ed.

Skaar C (1984) Wood–water relationships in the chemistry of solid wood. In *The Chemistry*

*of Solid Wood, Vol. 207*, (American Chemical Society), pp 127–172.

Sprung M. (1984). Physiological energetics of mussel larvae (*Mytilus edulis*). I. Shell growth

and biomass. *Marine ecology progress series. Oldendorf*, *17*(3), 283-293.

Stoeckel JA, Padilla DK, Schneider DW & Rehmann CR (2004). Laboratory culture of

*Dreissena polymorpha* larvae: spawning success, adult fecundity, and larval mortality patterns. *Canadian Journal of Zoology*, *82*(9), 1436-1443.

Wadsö L (1994) Describing non-Fickian water-vapour sorption in wood. *Journal of*

*Materials Science* 29(9):2367–2372.

Wang H*, et al.* (2008) Modeling oyster growth rate by coupling oyster population and

hydrodynamic models for Apalachicola Bay, Florida, USA. *Ecological Modelling* 211(1-2):77–89.

Wiegand T, Gunatilleke S, & Gunatilleke N (2007) Species associations in a heterogeneous

Sri Lankan dipterocarp forest. *The American Naturalist* 170(4):E77–E95.

Wood J & Gladden LF (2002) Modelling diffusion and reaction accompanied by capillary

condensation using three-dimensional pore networks. Part 1. Fickian diffusion and pseudo-first-order reaction kinetics. *Chemical Engineering Science* 57:3033–3045.

Wood J, Gladden LF, & Keil FJ (2002) Modelling diffusion and reaction accompanied by

capillary condensation using three-dimensional pore networks. Part 2. Dusty gas

model and general reaction kinetics. *Chemical Engineering Science* 57(15):3047–3059.
