# Supplementary figures and images for: Reconstructing the ecology of a Jurassic pseudoplanktonic raft colony
Source: R Soc Open Sci. 2020 Jul 22;7(7):200142. doi: 10.1098/rsos.200142 (PMC7428219; doi:10.1098/rsos.200142)

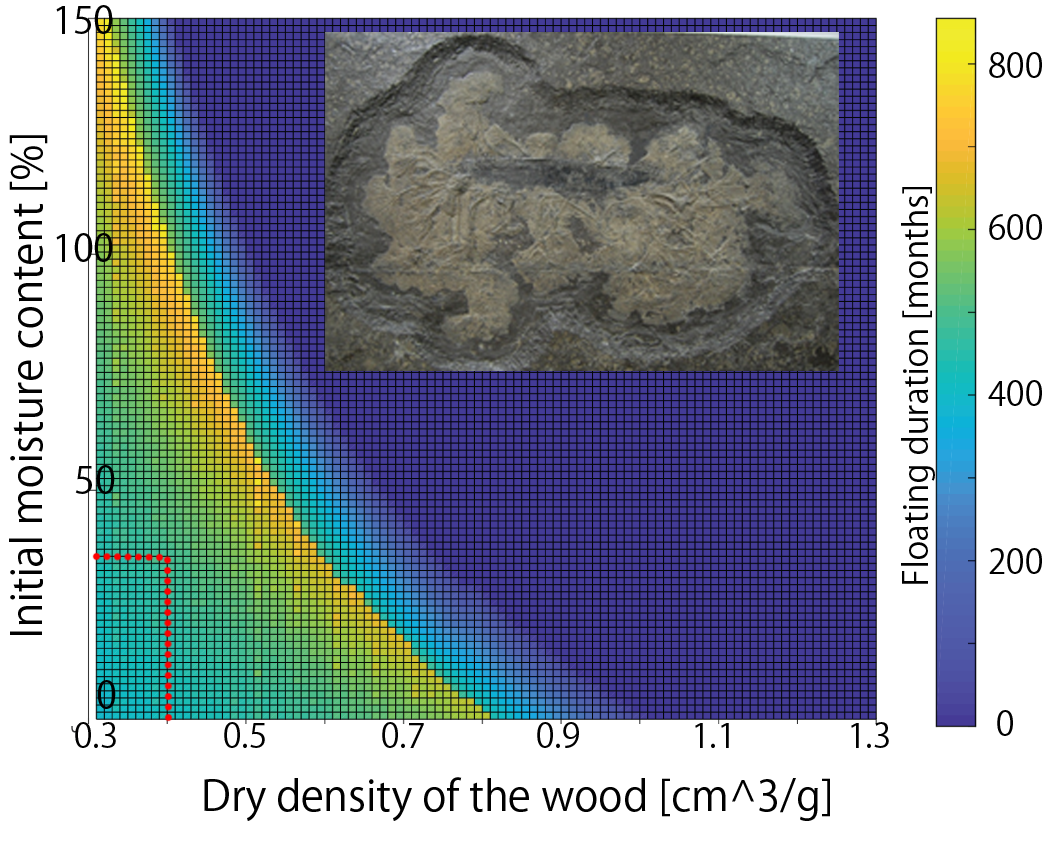

Supplement: Figure S4 [file rsos200142supp2.tif]

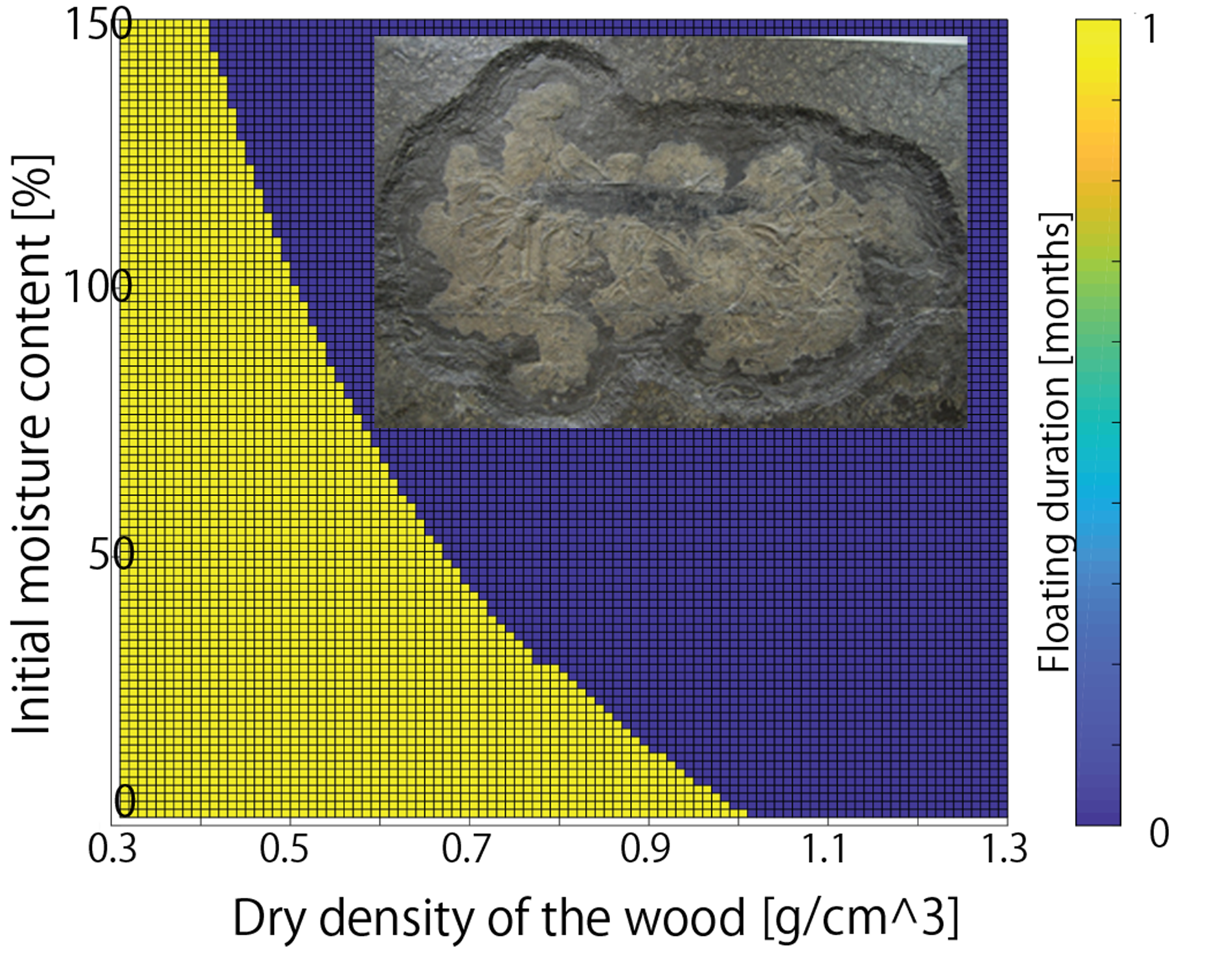

Supplement: Figure S5 [file rsos200142supp3.tif]

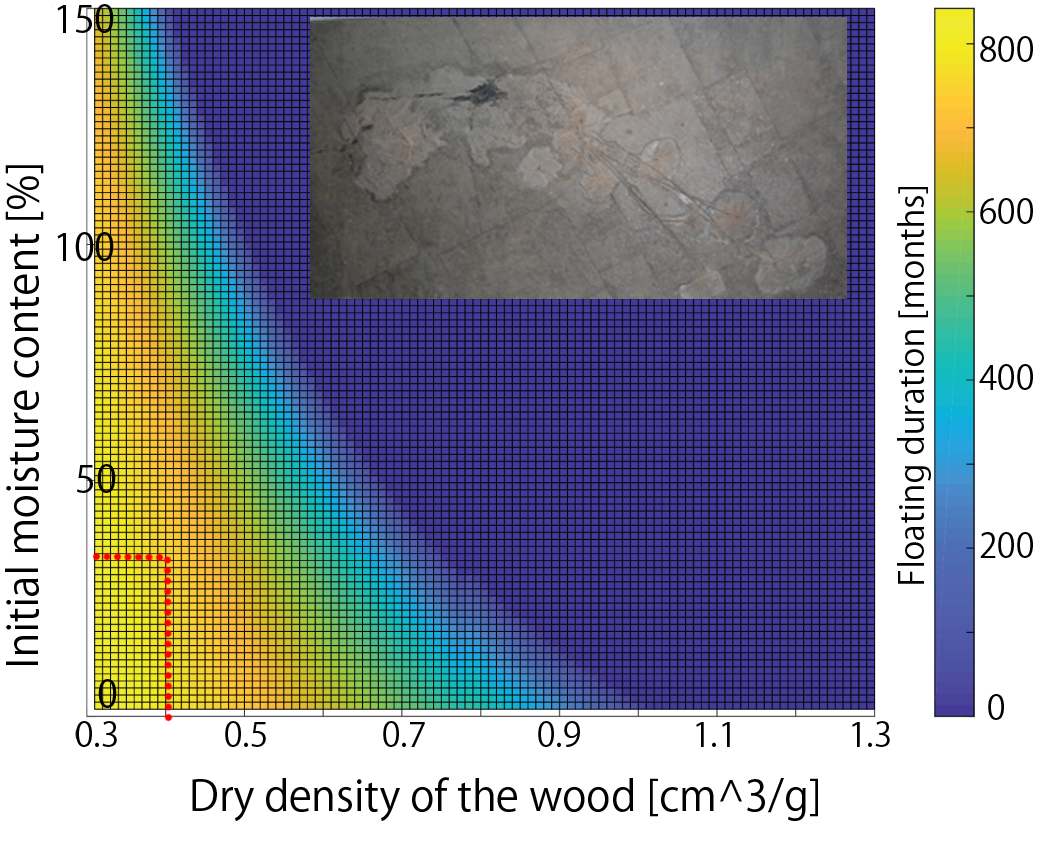

Supplement: Figure S6 [file rsos200142supp4.tif]

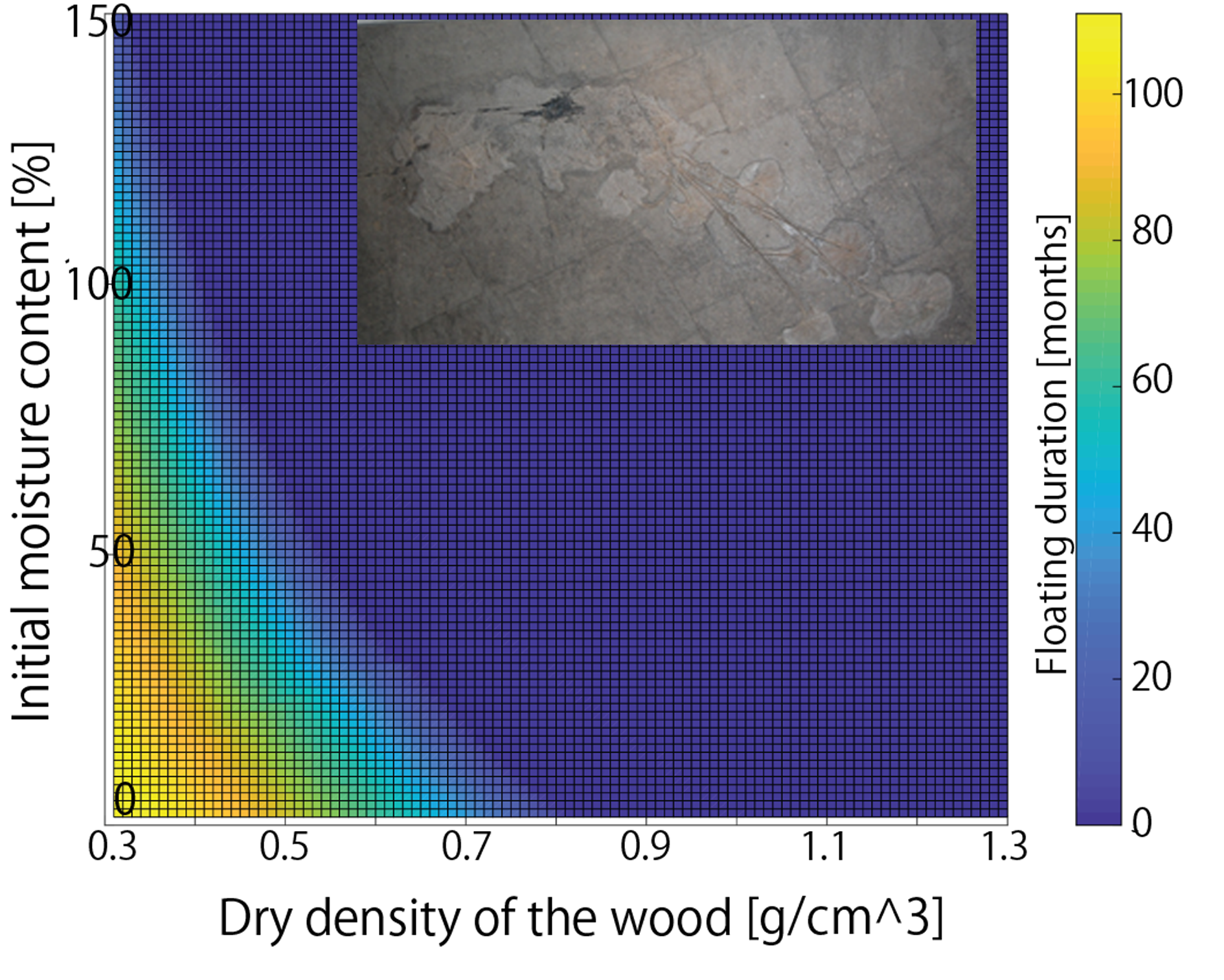

Supplement: Figure S7 [file rsos200142supp5.tif]

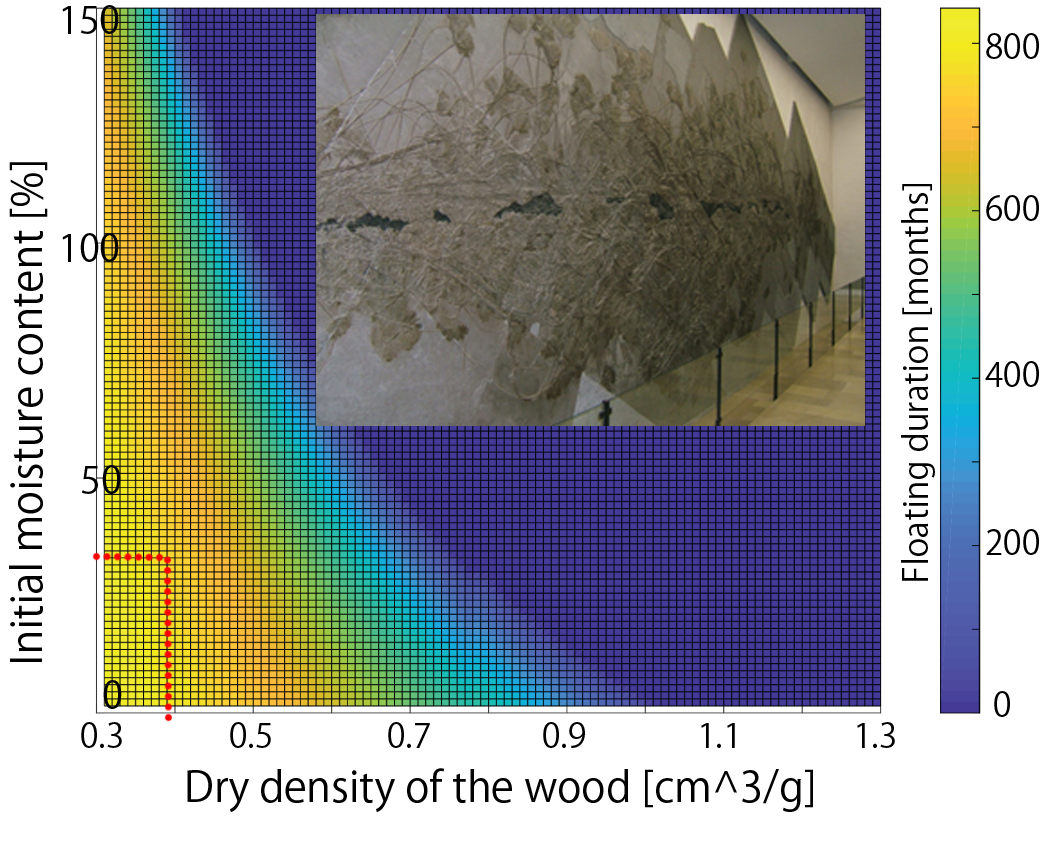

Supplement: Figure S8 [file rsos200142supp6.tif]

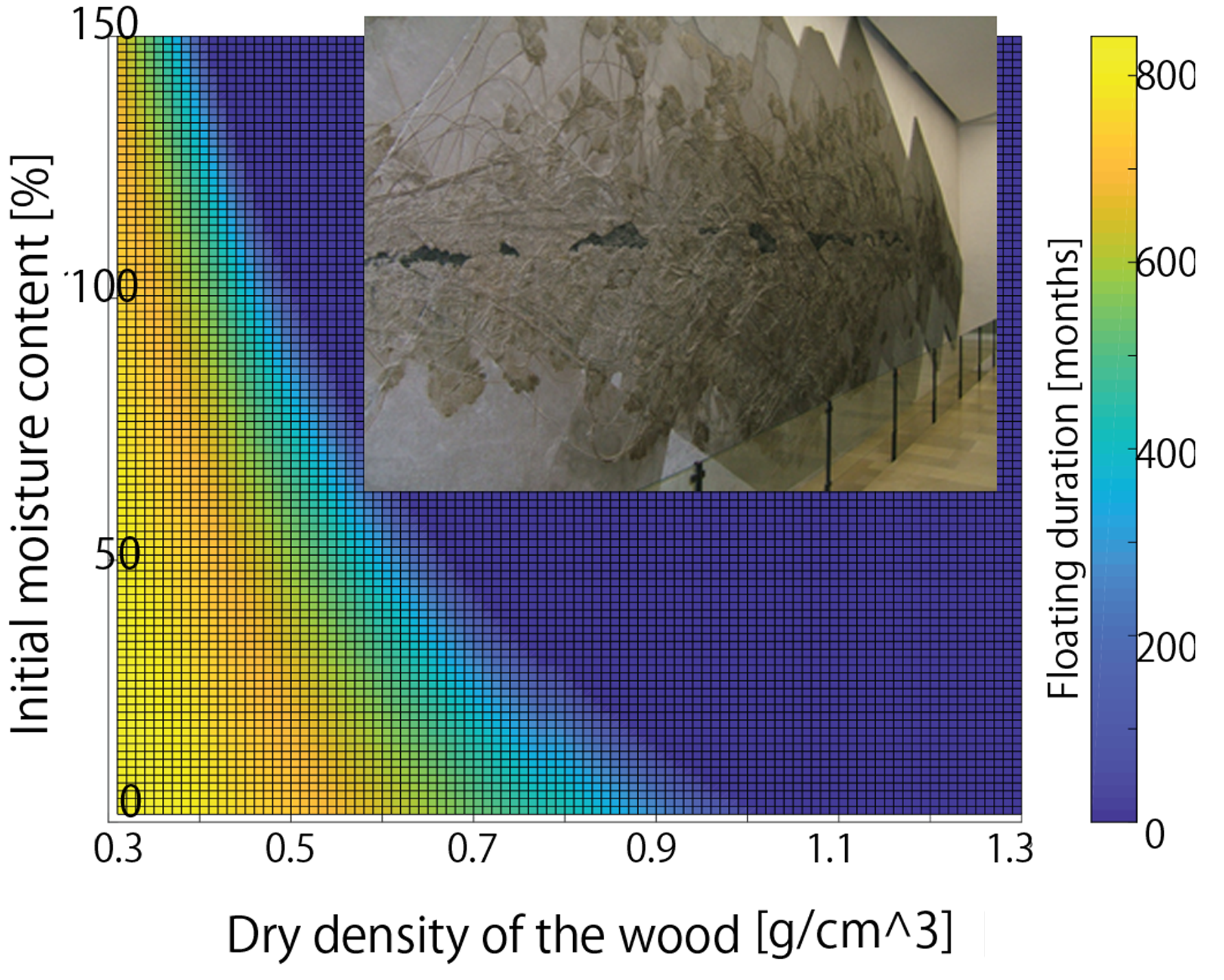

Supplement: Figure S9 [file rsos200142supp7.tif]
